# Invading a mutualistic network: to be or not to be similar

**DOI:** 10.1002/ece3.2263

**Published:** 2016-06-23

**Authors:** Henintsoa Onivola Minoarivelo, Cang Hui

**Affiliations:** ^1^ Centre for Invasion Biology Department of Mathematical Sciences Stellenbosch University Matieland 7602 South Africa; ^2^ Centre of Excellence in Mathematical and Statistical Sciences Wits University Gauteng 2050 South Africa; ^3^ Theoretical Ecology Group African Institute for Mathematical Sciences Cape Town 7945 South Africa

**Keywords:** Disruptiveness, invasibility, invasiveness, mutualistic networks, resilience, robustness

## Abstract

Biological invasion remains a major threat to biodiversity in general and a disruptor to mutualistic interactions in particular. While a number of empirical studies have directly explored the role of invasion in mutualistic pollination networks, a clear picture is yet to emerge and a theoretical model for comprehension still lacking. Here, using an eco‐evolutionary model of bipartite mutualistic networks with trait‐mediated interactions, we explore invader trait, propagule pressure, and network features of recipient community that contribute importantly to the success and impact of an invasion. High level of invasiveness is observed when invader trait differs from those of the community average, and level of interaction generalization equals to that of the community average. Moreover, multiple introductions of invaders with declining propagules enhance invasiveness. Surprisingly, the most successful invader is not always the one having the biggest impact on the recipient community. The network structure of recipient community, such as nestedness and modularity, is not a primary indicator of its invasibility; rather, the invasibility is best correlated with measurements of network stability such as robustness, resilience, and disruptiveness (a measure of evolutionary instability). Our model encompasses more general scenarios than previously studied in predicting invasion success and impact in mutualistic networks, and our results highlight the need for coupling eco‐evolutionary processes to resolve the invasion dilemma.

## Introduction

Rapid global changes induced by anthropogenic disturbance constitute a major threat to networks of ecological interactions (Tylianakis et al. 2008; Burkle and Alarcón 2011), of which biological invasion represents one important component (Morales and Traveset 2009; McGeoch et al. 2010). Mutualistic networks of pollination and seed dispersal are key service providers in ecosystems (Bronstein 2001); understanding how their structures and stabilities respond to biological invasions is paramount to safeguarding ecosystem function and service in a changing world (Traveset and Richardson 2006; Lurgi et al. 2014; Campbell et al. 2015). For efficient prevention and control, the challenge is to foresee the invasiveness and impact of potential invaders in given ecosystems. This is a challenge of complexity as no universal rules, except for the amount of propagules introduced (known as the propagule pressure; Williamson, 1999; Jeschke and Strayer 2006; Simberloff 2009), govern the process and success of invasion which are nearly exclusively contingent on the taxa and context (Williamson and Fitter 1996).

When introduced into a new environment, an alien species needs to compete for space and resources with native resident species, simply by possessing certain phenotypic and behavioral traits (Romanuk et al. 2009). The strength of ecological interactions is often mediated by matching between functional traits of interacting species (Jousselin et al. 2003; Santamaría and Rodríguez‐Gironés 2007; Stang et al. 2009). A certain degree of similarity between the trait of invasive and resident species often indicates a strong mutualistic interaction (Gibson et al. 2012). Nevertheless, species with high invasiveness and impact in pollination networks acquire traits atypical of native (Aizen et al. 2008; Campbell et al. 2015; but see Morales and Traveset 2009). As such, features of both invaders and recipient communities play critical roles in predicting the success and impact, two interdependent elements, of an invasion (Shea and Chesson 2002; Gurevitch et al. 2011).

Such interdependence of invasiveness and impact could be further amplified in an ecological network because of cascading interactions (Bascompte and Stouffer 2009; Dunne and Williams 2009; Traveset and Richardson 2014). Species with a high level of interaction generalization, that is, high‐degree nodes in a network, has been shown to determine the invasion success in both food webs (Romanuk et al. 2009; Lurgi et al. 2014) and mutualistic networks (Traveset and Richardson 2014). Functional traits, such as body size and diet breadth that are indicative to species' trophic position in a food web and thus its level of interaction generalization, are good predictors of invasion success. For instance, consumer species with a wide diet breadth or a large body size experience more invasion success in a food web (Lurgi et al. 2014). Invasive plants in pollination networks often have higher levels of interaction generalization than natives (Albrecht et al. 2014). The overall interactions in a pollination network can even be monopolized by super‐generalist invaders (Aizen et al. 2008; Bartomeus et al. 2008; Vilà et al. 2009).

Characteristics of a recipient ecosystem responsible for its susceptibility to the establishment and spread of invasive species defines its invasibility (Lonsdale 1999; Alpert et al. 2000). Besides physical factors such as habitat suitability and heterogeneity, other major characteristics considered in literature include the network architecture of biotic interactions. For example, a high level of network connectance – the proportion of realized interactions among possible ones – has been predicted to enhance the resistance of food webs to invasion (Romanuk et al. 2009), although contested by others (Baiser et al. 2010; Lurgi et al. 2014). Modularity – the extent to which a network is organized into groups of species interacting more strongly with species from the same group rather than from other groups – is observed to be lower in invaded pollination networks and food webs than in uninvaded ones (Albrecht et al. 2014; Lurgi et al. 2014). Empirical studies have also revealed that invaded pollination networks are more nested – where specialists interact only with a subset of species with which generalists interact – and normally contain a higher number of species than uninvaded networks (Padrón et al. 2009; Stouffer et al. 2014).

Mutualistic interactions normally have a facilitative effect on the establishment of alien species (Traveset and Richardson 2014). Successful invaders in mutualistic networks have been shown to interact with either the most specialist natives (Stouffer et al. 2014) or the most generalist ones (Padrón et al. 2009). However, empirical observations do not allow for discerning whether some network features could have triggered the invasion or are indeed resulting from the invasion. By comparing the pre‐ and postinvasion architectures of simulated pollination networks, Campbell et al. (2015) managed to fill the gap in literature and found that, while network connectance decreased, nestedness increased from invasions.

The role of particular network architectures in stabilizing networks has been hotly debated, especially regarding mutualistic networks. On one hand, patterns of connectance and nestedness observed in mutualistic networks can facilitate the coexistence of species and thus contribute positively to network stability (Bastolla et al. 2009; Thébault and Fontaine 2010; Rohr et al. 2014). Network complexity, measured as network size and connectivity (number of interactions), can enhance network resilience (Okuyama and Holland 2008). On the other hand, some theoretical studies have shown that these typical features specific to mutualistic networks can also be detrimental to network stability. For instance, the stability of a mutualistic network declines with extreme levels of nestedness (Campbell et al. 2012) or modularity (Thébault and Fontaine 2010). The stability of a mutualistic network was also found to be negatively correlated with connectance especially when interaction strength is taken into account (Allesina and Tang 2012; Vieira and Almeida‐Neto 2015).

Inconsistency of the correlation between network structure and network stability is somewhat caused by the confusion in choosing appropriate measures of network stability. Each metric of network stability only measures one specific facet of stability and thus often leads to contradictions when interpreted as the general stability for comparison (Vallina and Quéré 2011). Among these metrics of network stability/instability, network invasibility is a recent emergent concept particularly relevant to invasion biology; it is defined as the amount of opportunity niches in the trait space that allow for positive per‐capita population growth of rare aliens (Hui et al. 2016). It is therefore necessary to explore how the concept of invasibility relates to these other measures of network stability/instability, as well as how these stability measures (including invasibility) are correlated with network architectures and the invasiveness of aliens.

Although the literature in invasion ecology is dominated by empirical and experimental studies, theoretical works are needed to explore general rules for predicting invasiveness and impacts of alien species. Models with trait‐mediated biotic interactions represent an ideal theoretical framework for exploring issues of biological invasion. For example, Campbell et al. (2015) formulated the interaction strength between newly introduced species and resident species by the similarity between their phenotypic traits such as between corolla depth of plants and proboscis length of pollinators. In these studies, traits of resident species are static and either randomly assigned (Romanuk et al. 2009; Lurgi et al. 2014) or empirically inferred (Campbell et al. 2015). However, resident traits are often adaptive and results from long‐term ecological and evolutionary processes. The role of such adaptive nature of resident traits in invaded networks needs to be assessed. Here, we deploy a theoretical approach to explore the process of biological invasion in mutualistic networks. Mutualistic networks are described using an eco‐evolutionary model depicting simultaneously ecological dynamics of population densities happening at a faster timescale and evolutionary dynamics of functional traits happening at a slower timescale, using the framework of *adaptive dynamics* (Metz et al. 1992; Dieckmann and Law 1996). In these networks, each species is identified by its trait (i.e., as morphospecies) which determines the intensity of both intraspecific competition and mutualistic interaction. Our previous work using a similar model has shown that properties of emerged mutualistic networks are comparable to features of empirical networks (Minoarivelo and Hui 2016). However, we did not explore how an introduced species performs and how emerged mutualistic networks, in terms of their architectures and stability, respond to the incursions of these introduced species. Here, we first use the model to generate mutualistic networks as recipient communities, into which we then introduce an alien species. By examining a wide range of possibilities for both invaders and recipient communities, we investigate how they respond to each other. In particular, we study (1) how the invasiveness and the impact of an introduced species depend on whether or not its trait and its level of interaction generalization are relatively similar to the average of the recipient community; (2) how the success of an invasion depends on the way the invasive species is introduced, that is, propagule pressure; and (3) how the invasibility and other metrics of network stability depend on the structure of recipient communities.

## Materials and Methods

Evolutionary and ecological processes are coupled. Evolutionary changes in functional traits can affect ecological processes such as the way species interact with each other and subsequently the behavior of population dynamics and demography (Hui et al. 2015). In return, functional traits evolve in response to varying frequency‐dependent selection from changing population densities. As such, we design a model of mutualistic network emergence, implementing exactly such coupling of population dynamics and trait evolution. Specifically, we assume that resource competition becomes intense when the two species involved have similar traits, as illustrated in the limiting similarity theory stating the existence of a threshold for the similarity between two species above which coexistence cannot be guaranteed due to competitive exclusion (Abrams 1983). We also assume that matching traits between a pair of mutualistically interacting animal and plant species (i.e., assortative interactions) can expect high fitness rewards. For pollination syndromes, pollinator trait could be its proboscis length, and floral trait could be the length of pollen tube. For seed dispersal syndromes, traits could be the body size of animal dispersers or the fruit size of the plant. Following the framework of adaptive dynamics, traits can evolve either directionally or disruptively, and the latter case allows a single trait to diversify adaptively into two, eventually forming an ecological network. The resultant network from such trait evolution will be considered as a resident native mutualistic network into which we introduce an alien species. We generate multiple mutualistic networks with different characteristics to explore the role of network architectures in resisting invasions. We further vary the trait value of the introduced species to examine the potential characteristics of a successful invader.

### Ecological dynamics

Let there be *n* morphospecies of animals and *m* morphospecies of plants. Each morphospecies, indexed by *i* for animals and *j* for plants, is further characterized by its population density *A*_*i*_ (for *i* ∈ 1, … ,*n*) and *P*_*j*_ (for *j* ∈ 1, …, *m*), respectively. We denote the trait of animal morph *i* by *x*_*i*_ and the trait of plant morph *j* by *y*_*j*_. The population dynamics of the system is governed by the per‐capita population growth rates, dependent on the intrinsic growth rate, intratrophic competition, and cross‐trophic mutualistic interactions (following Holling's type II functional response (1959)) (Holland et al. 2006; Zhang et al. 2011; Nuwagaba et al. 2015; Minoarivelo and Hui 2016):

(1a) $$\frac{dA_{i}}{A_{i}dt}=f_{A}\left(x_{i}\right)=r_{A}-\frac{r_{A}\sum_{k}\gamma \left(x_{i},x_{k}\right)A_{k}}{K_{A}\left(x_{i}\right)}+\frac{\sum_{j}b_{A_{i}P_{j}}w_{A_{i}P_{j}}P_{j}}{1+h\sum_{j}w_{A_{i}P_{j}}P_{j}}$$

(1b) $$\frac{dP_{j}}{P_{j}dt}=f_{P}\left(y_{j}\right)=r_{P}-\frac{r_{P}\sum_{k}\gamma \left(y_{j},y_{k}\right)P_{k}}{K_{P}\left(y_{j}\right)}+\frac{\sum_{i}b_{P_{j}A_{i}}w_{P_{j}A_{i}}A_{i}}{1+h\sum_{i}w_{P_{j}A_{i}}A_{i}}$$

where *r* is the intrinsic population growth rate, and *h* the handling time that animals spend for visiting a plant and digesting the nutrients extracted from the plant; both are assumed to be trait‐independent to avoid overparameterization of the model (*r*_*A*_ = *r*_*P*_ = 1; *h *=* *0.1). Note that parameter values provided below in brackets are used as reference for sensitivity tests. In the following, all terms in eq. (1b) can be mirrored from the specified formulation in eq. (1a). Descriptions of all parameters in eqs. (1a) and (1b) are summarized in Table 1.

**Table 1:** A summary of model parameters

Parameter	Description
*A*_*i*_, *P*_*j*_	Population density
*x*_*i*_, *y*_*j*_	Trait value
*r*_*A*_, *r*_*P*_	Intrinsic population growth rate
*K*_*A*_(*x*_*i*_), *K*_*P*_(*y*_*j*_)	Carrying capacity, functions of the trait value
*γ*(*x*_*i*_, *x*_*k*_), *γ*(*y*_*j*_, *y*_*k*_)	Intratrophic competition, functions of the trait values of two involved species
$b_{A_{i}P_{j}},b_{P_{j}A_{i}}$	Cross‐trophic mutualistic benefit, functions of the trait values of two involved species
$w_{A_{i}P_{j}},w_{P_{j}A_{i}}$	Interaction preference after encounter, functions of both mutualistic benefit and population abundance
*h*	Handling time

The carrying capacity, *K*_*A*_ and *K*_*P*_, varies between morphs, representing trait‐mediated resource accessibility. Following Doebeli and Dieckmann (2000), we used a Gaussian function for the carrying capacity:

(2) $$K_{A}\left(x_{i}\right)=k_{A}N\left(x_{A}^{\max},\sigma_{A},x_{i}\right)$$

where *k*_*A*_ (=400) is a scaling constant, and $N(x_{A}^{\max},\sigma_{A},x_{i})$ the Gaussian density function of trait *x*_*i*_ with the maximum carrying capacity at $x_{A}^{\max}$ (=3) and the standard deviation *σ*_*A*_. This means that there exists an optimal trait value for accessing resources at a maximum level *k*_*A*_. Species with trait deviating from the optimal trait suffer from lower resource accessibility and thus lower carrying capacity. Similarly, we set the baseline values of *k*_*P*_ (=300) and $y_{P}^{\max}$ (=2) for the plant species in the following analysis.

The intratrophic competition function *γ* is set to let morphs with more similar traits suffer stronger competition. We used a Gaussian function for depicting the competition intensity between morphs (Doebeli and Dieckmann 2000; Bürger et al. 2006; Raimundo et al. 2014):

(3) $$\gamma \left(x_{i},x_{k}\right)=\exp\left(-{\left(x_{i}-x_{k}\right)}^{2}/2\sigma_{C}^{2}\right)$$

where *σ*_*C*_ controls the width of the competition kernel. This means that intratrophic competition becomes less sensitive to trait difference between the two competing species as the width of competition kernel *σ*_*C*_ becomes larger. In such a case, species can compete with a wider range of other species for resources.

The cross‐trophic mutualistic benefit, *b*_*AP*_, reflects the assumption of assortative interactions that matched traits bring to each other high profit and is also assumed to follow a Gaussian function of trait difference:

(4) $$b_{AP}\left(x_{i},y_{j}\right)=c\cdot \exp\left(-{\left(x_{i}-y_{j}\right)}^{2}/2\sigma_{m}^{2}\right)$$

where *c* (=0.1) is a parameter controlling the magnitude of the maximum mutualistic support, and the parameter *σ*_*m*_ controls the tolerance level of successful interactions to the dissimilarity of involved traits (Nuismer et al. 2010). This means that a species having trait value similar to its mutualistic partner gains the highest mutualistic benefit. As the tolerance level to trait difference (*σ*_*m*_) becomes smaller, mutualistic benefits can only be assured for partners having very similar traits. A high level of tolerance to trait difference means that partner species with dissimilar traits can also gain rewards from their mutualistic interactions.

The interaction preference of two morphs *w*_*AP*_ determines the possibility of interaction after the encounter and is assumed to follow adaptive foraging strategies, depending on both the benefit and abundance of involved morphs (Doebeli and Dieckmann 2000; Zhang and Hui 2014). Modifying the expression which describes the strength of mutualistic support in Doebeli and Dieckmann (2000), we have the following function for the adaptive interaction preference:

(5) $$w_{A_{i}P_{j}}=\frac{b_{A_{i}P_{j}}^{}\Sigma_{k}A_{k}}{\Sigma_{k}A_{k}b_{A_{k}P_{j}}^{}}$$

where the summation term Σ_*k*_ *A*_*k*_ in the numerator is for normalization. This means that an animal prefers to interact with plants that are common and with matching traits.

### Evolutionary dynamics

Functional traits of interacting morphs are subject to mutations. This can also be interpreted as the replacement and reassembling of local species through colonization of regional species with different traits to these local residents. Mutation normally happens at a low rate so that the populations can be considered at their ecological equilibriums when the mutation occurs (Geritz et al. 1998). We only consider the nontrivial strictly positive and asymptotically stable equilibrium points of the system (${\tilde{A}}_{i}\left(x_{i},y_{j}\right)$ and ${\tilde{P}}_{j}\left(x_{i},y_{j}\right)$). When a mutation enters the system, the resident morphospecies and the mutant undergo an intratrophic competition determined by eq. (1). Let $x_{i}^{\prime }$ and $y_{i}^{\prime }$ be the mutant trait of animal morphospecies *i* and plant morphospecies *j*, and let *X* = (*x*_1_, …, *x*_*n*_) and *Y* = (*y*_1_, …, *y*_*m*_) be the trait vectors of the resident morphospecies. We can define the invasion fitness of the rare mutants at the equilibrium points as their per‐capita growth rates when setting their initial densities to be negligible: *f*_*A*_($x_{i}^{\prime }$) and *f*_*P*_($y_{j}^{\prime }$). The selection gradient, defined as,

(6) $$\begin{array}{cc} & g_{A_{i}}=\partial f_{A}\left(x_{i}^{\prime }\right)/\partial x_{i}^{\prime }{|}_{x_{i}^{\prime }=x_{i}}\\ & g_{P_{j}}=\partial f_{P}\left(y_{j}^{\prime }\right)/\partial y_{j}^{\prime }{|}_{y_{j}^{\prime }=y_{j}} \end{array}$$

determines the direction and speed of trait evolution, and an evolutionary singularity is defined as the traits $\left({\tilde{x}}_{i},{\tilde{y}}_{j}\right)$ when the selection gradient disappears.

The evolutionary dynamics of the functional traits can be depicted by the canonical equations of adaptive dynamics (Dieckmann and Law 1996):

(7) $$\begin{array}{cc} & dx_{i}/dt=m_{A}{\tilde{A}}_{i}g_{A_{i}}\\ & dy_{j}/dt=m_{P}{\tilde{P}}_{j}g_{P_{j}} \end{array}$$

where *m*_*A*_ and *m*_*P*_ are parameters proportional to the rate and variation of the mutation (set to 10^−3^) in the analysis. An evolutionary branching is to occur in the system provided that three conditions are satisfied. First, the singularity $\left({\tilde{x}}_{i},{\tilde{y}}_{j}\right)$ should be an evolutionary attractor of directional selection; that is, it is convergence stable. This happens when all eigenvalues of the Jacobian matrix of eq. (7) have negative real parts (see Doebeli and Dieckmann 2000); this means:

(8) $$\begin{array}{cc} & \partial g_{A_{i}}/\partial x_{i}{|}_{x_{i}={\tilde{x}}_{i}}<0\\ & \partial g_{P_{j}}/\partial y_{j}{|}_{y_{j}={\tilde{y}}_{j}}<0 \end{array}$$

Second, the singularity should represent a fitness minimum to induce disruptive selection and to allow the mutant to invade (Geritz et al. 1998); that is,

(9) $$\begin{array}{cc} & \partial^{2}f_{A}/\partial {x_{i}^{\prime }}^{2}{|}_{x_{i}^{\prime }={\tilde{x}}_{i}}>0\\ & \partial^{2}f_{P}/\partial {y_{j}^{\prime }}^{2}{|}_{y_{j}^{\prime }={\tilde{y}}_{j}}>0 \end{array}$$

Finally, the mutant and the resident morphospecies need to coexist to insure the protection of dimorphism from the evolutionary branching (Geritz et al. 1998); that is, the two morphospecies can invade each other:

(10) $$\begin{array}{cc} & \left(\partial^{2}f_{A}/\partial {x_{i}}^{2}+\partial^{2}f_{A}/\partial {x_{i}^{\prime }}^{2}\right){|}_{x_{i}^{\prime }=x_{i}=\tilde{x}}>0\\ & \left(\partial^{2}f_{P}/\partial y_{j}^{2}+\partial^{2}f_{P}/\partial {y^{\prime }}_{j}^{2}\right){|}_{y_{j}^{\prime }=y_{j}={\tilde{y}}_{j}}>0 \end{array}$$

### Numerical analysis

We numerically solved the population dynamics (eq. 1) and the canonical equations of adaptive dynamics (eq. (7)). It is worth noting that, although the trait of a species can take any values (e.g., log‐transformed body size as a focal trait can range from negative to positive infinity, theoretically speaking), only those that are feasible and can insure its own viability, that is, with a positive equilibrium in eq. (1), can be realized in the model. Once the system reaches its singularity (i.e., when directional selection ceases, with populations also at the ecological equilibrium), the three conditions for evolutionary branching will be examined. If satisfied, a new morphospecies will be added to the system with its trait value slightly different from the resident trait (+0.01) and having a low initial density (10% of its resident population density). The density of the resident morphospecies will be simultaneously updated to be 90% of its original. The process was repeated until we obtain adequate number of morphospecies to form a network and the system has reached its singularity. A morphospecies was considered extinct when its population density dropped below 10^−8^.

**Figure 1:**
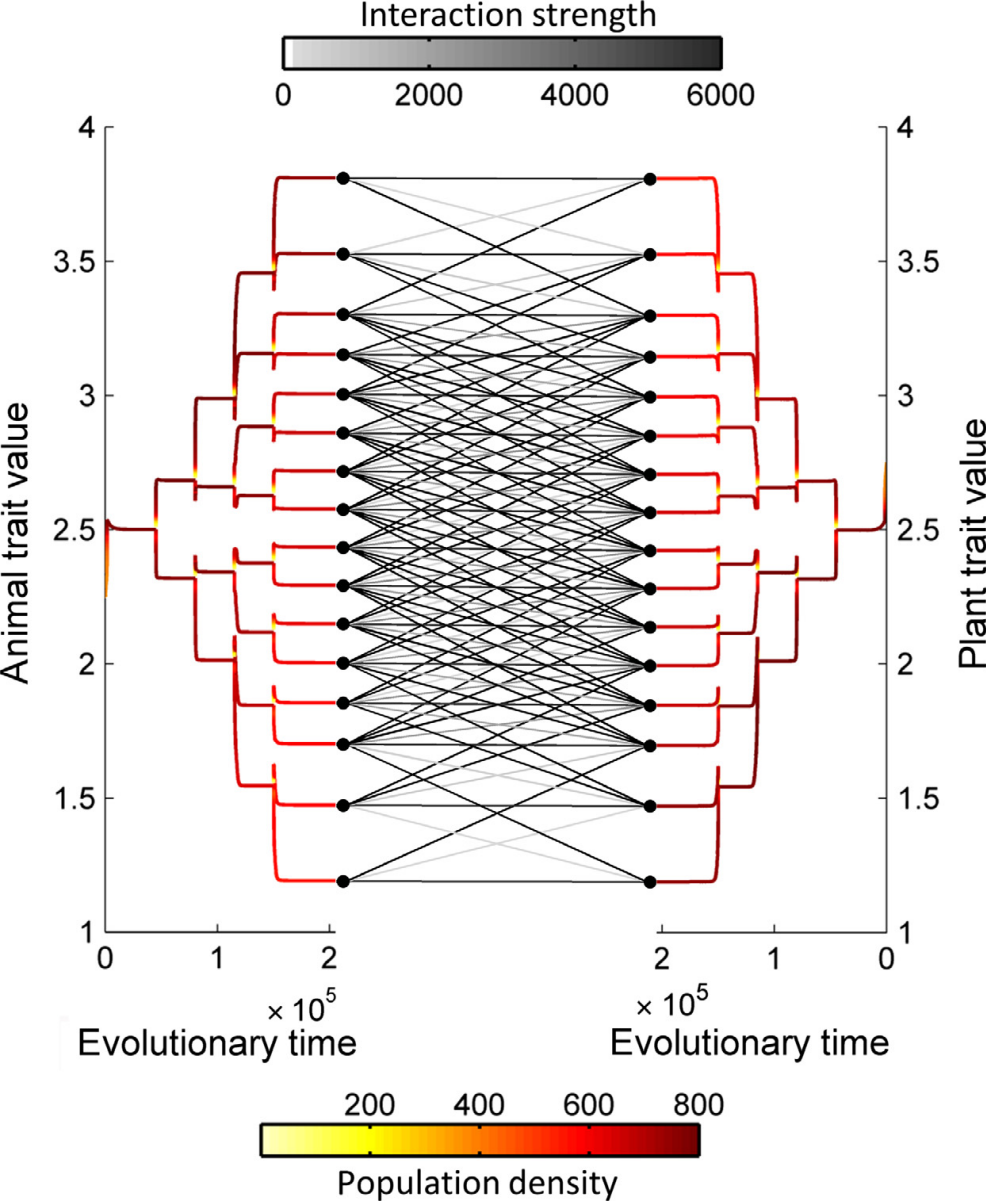
Evolutionary dynamics of a mutualistic network. The trait dynamics preinvasion is represented as two evolutionary trees and its associated interaction network represented as a bipartite graph. Parameters: *σ*_*A*_ = e^0.75^; *σ*_*c*_ = e^−3^; *σ*_*m*_ = e^−2.25^.

We distinguished three types of communities depending on their sizes. Small communities were generated by allowing the system to branch four consecutive times, giving a maximum number of 16 (=2^4^) morphs on each side of animals and plants. An example of the formation of a small community by trait evolution depicted as evolutionary trees is given in Figure 1. Medium‐size communities were generated by five consecutive branching events, giving a maximum of 32 (=2^5^) morphs on each side. Large communities were obtained by six consecutive branching events with a maximum of 64 (=2^6^) morphs on each side. We obtained communities with different structures by varying kernel parameters (Minoarivelo and Hui 2016): the width of the intratrophic competition kernel (*σ*_*C*_), the tolerance to trait difference in a mutualistic interaction (*σ*_*m*_), and the width of resources accessibility (*σ*_*A*_ for animals and *σ*_*P*_ for plants; we keep *σ*_*A*_ = *σ*_*P*_ for simplicity). These parameters were varied from *e* ^−3^ (≈0.05) to *e*, with a multiplicative step of e^1/4^. We discarded the combinations of *σ*_*C*_, *σ*_*m,*_ and *σ*_*A*_ that resulted in monomorphic systems (no diversification).

### Network analysis

We considered the bipartite mutualistic networks formed by interactions between the two sets of animal and plant morphospecies. Here, we depicted the network as a quantitative interaction matrix (*Q*) where its elements (*q*_*ij*_) represent the interaction strength between animal *i* and plant *j*. Following Berlow et al. (2004), we define the interaction strength as the nonlinear functional response term of eq. (1), depending on both the number of recruited animal *i* from interacting with plant *j* and the number of recruited plant *j* from interacting with animal *i*, per time unit:

(11) $$q_{ij}=\frac{1}{2}\left(\frac{A_{i}b_{A_{i}P_{j}}w_{A_{i}P_{j}}P_{j}}{1+hw_{A_{i}P_{j}}P_{j}}+\frac{P_{j}b_{P_{j}A_{i}}w_{P_{j}A_{i}}A_{i}}{1+hw_{P_{j}A_{i}}A_{i}}\right)$$

When the element *q*_*ij*_ is less than 10^−8^, it was considered to be equal to zero, indicating a negligible interaction. An illustration of such interaction network as a bipartite weighted graph is given in Figure 1.

We analyzed the architecture of the networks using four metrics adapted for quantitative matrices. First, the level of interaction specialization (SPE) of each network was measured according to the quantitative index $H_{2}^{\prime }$ of Blüthgen et al. (2006). This index measures the overall deviation of species' realized degrees from their expected ones, ranging from 0 (no specialization) to 1 (perfect specialization). Second, the quantitative connectance metric (CON) was computed as the quantitative linkage density (i.e., the mean number of interactions per species, weighted by interaction strength) divided by the number of species in the network (Tylianakis et al. 2007). This index is directly related to the proportion of realized interactions in the network when interaction strengths are taken into account. Third, we used the metric WNODF (weighted nestedness metric based on overlap and decreasing fill) for depicting the level of nestedness (NEST) (Almeida‐Neto and Ulrich 2011). The WNODF metric is based on the assumption that if species *i* is more specialized than species *j*, then the interaction between species *i* and *k* will only be counted when species *j* also interacts with species *k*. Finally, the level of modularity was measured using the algorithm *QuanBimo* (Dormann and Strauβ 2014). By assuming that the average interaction strength within a module is higher than between modules, the *Quanbimo* algorithm forms a module by assigning species that interact more strongly with species within the module than expected by chance. All these network metric measurements are implemented in the R library *bipartite* (Dormann et al. 2008).

### Invasion trial

As the model is symmetric regarding animals and plants side, we introduced an alien animal species into the native community, with the number of individuals introduced equal to 5%, 10%, and 25% of the average population density in the recipient community. Because effects of biological invasion are generally studied at ecological timescales, we fixed the phenotypic traits of the studied community once the alien species was introduced and only allowed population densities to change according to eq. (1).

To test the dependence of invasion success on the particular ways that these propagules were introduced, we randomly selected 100 medium‐size networks and tested five different ways of introducing the alien propagules. First, all individuals of the alien species were introduced only once before letting population dynamics to change. Second, individuals of the alien species were divided into two groups of equal size. The first group was introduced at the initial time step while the second group after five time steps. Third, individuals of the alien species were introduced at three consecutive times separated by an interval of five time steps. The number of individuals introduced increased each time, representing 20%, 30%, and 50% of the total propagule size. Fourth, individuals were introduced three times but with declining numbers each time (50%, 30%, and 20%). Finally, we introduced the alien species five consecutive times with equal densities (20% each time), with introductions separated by five time steps.

We further investigated the role of the trait and the level of mutualism generalization of the invader, relative to the resident species in recipient communities. First, we introduced animal species with nine different trait values, ranging evenly from the lowest to the highest trait value of the natives. Hereafter, the trait value of the invader is reported as the relative trait value (rtv) and scaled between 0 (lowest trait value) to 1 (highest trait value), relative to the traits of resident species. Second, the level of mutualism generalization was measured as the tolerance of the invader to trait difference (i.e., *σ*_*m*_) for feasible mutualistic interactions. A high tolerance to trait difference (large *σ*_*m*_) suggests that mutualistic benefits can be assured by interacting with mutualistic partners with a wide range of traits, making the focal species a generalist. Nine levels of generalization of the invader were considered relative to the generalization level of the native community, with the generalization level ratio (glr) ranging from one‐fifth to five times the tolerance of native species to trait difference (*σ*_*m*_).

We considered two measurements of invasion success: invasiveness of the alien species, and the impact it has on the native community. Invasiveness (INVn) was defined as the relative growth rate of the invader: $\text{INVn}\;=\;\ln(A_{\mathrm{final}}^{\mathrm{inv}}/A_{\mathrm{initial}}^{\mathrm{inv}})$ in which $A_{\mathrm{final}}^{\mathrm{inv}}$ is the population density of the invader measured after the last possible introduction (i.e., at the 25th time step and after the fifth introduction which was at the 20th time step) and $A_{\mathrm{initial}}^{\mathrm{inv}}$ the total density of propagules introduced. The impact of the invasion (IMP) was measured as the magnitude of change in the relative growth rate of the native species: $\text{IMP}\;=\;|\ln(A_{\mathrm{final}}^{\mathrm{nat}}/A_{\mathrm{initial}}^{\mathrm{nat}})|$ in which $A_{\mathrm{final}}^{\mathrm{nat}}$ and $A_{\mathrm{initial}}^{\mathrm{nat}}$ denote the total population size of all native animals at the 25th time step and before the invasion, respectively. As the population dynamics in such models are fairly monotonic and smooth (e.g., see Okuyama and Holland 2008; Bastolla et al. 2009), measurements of invasiveness and impact at the 25th time step are sufficient to be indicative.

### Network stability and invasibility

To assess the potential ability of native communities to resist to biological invasions, we used a set of 1000 networks, including 350 small‐, 370 medium‐, and 280 large‐size networks. We calculated all commonly used stability metrics for these 1000 networks. First, network resilience (RES) was measured as the logarithm of the absolute value of the dominant eigenvalue of the Jacobian matrix at equilibrium (De Angelis 1980; Okuyama and Holland 2008; Encinas‐Viso et al. 2012): RES = ln |*λ*|. Specifically, the Jacobian matrix of the population dynamics (eq. 1) was computed at system singularity before alien introduction. Network resilience depicts how quickly a system returns to its steady state after being perturbed (De Angelis 1980). Second, we calculated network robustness (ROB) based on the concept of network response (secondary extinctions) from species removal (Dunne et al. 2002). Robustness is the fraction of species that had to be removed, from generalist to specialist, to result in the loss of more than 50% of all species. Finally, disruptiveness (DIS), a measure of evolutionary instability, was computed as the average of the strength of disruptive selection for all animal species (Brännström et al. 2011), with the strength of disruptive selection for a particular species *i* measured as the curvature of its invasion fitness at the singularity trait value ${\tilde{x}}_{i}$:

(12) $$\text{DIS}=\sum_{i=1}^{n}\partial^{2}f_{A}/\partial {x_{i}^{\prime }}^{2}{|}_{x_{i}^{\prime }={\tilde{x}}_{i}}.$$

We calculated the network invasibility (INVb) as the probability (proportion) of successful invasions (i.e., with positive invasiveness) among all invaders with traits spanning across the entire native trait range. We calculated the invasiveness and impact of an alien species when invading these 1000 networks. We assign each invader a trait as the average of native traits weighted by their population densities and a level of mutualistic generalization similar to the native community (glr = 1). We further assessed the relationship between network architecture (Network analysis) and stability metrics, including invasibility, using Spearman's rank correlation. We conducted a multidimensional scaling analysis of *k*‐mean clustering and hierarchical clustering (*pvclust* library in R, Suzuki and Shimodaira 2015) based on the rank correlation matrix to group closely related network metrics and observables.

## Results

### Role of invasive trait

**Figure 2:**
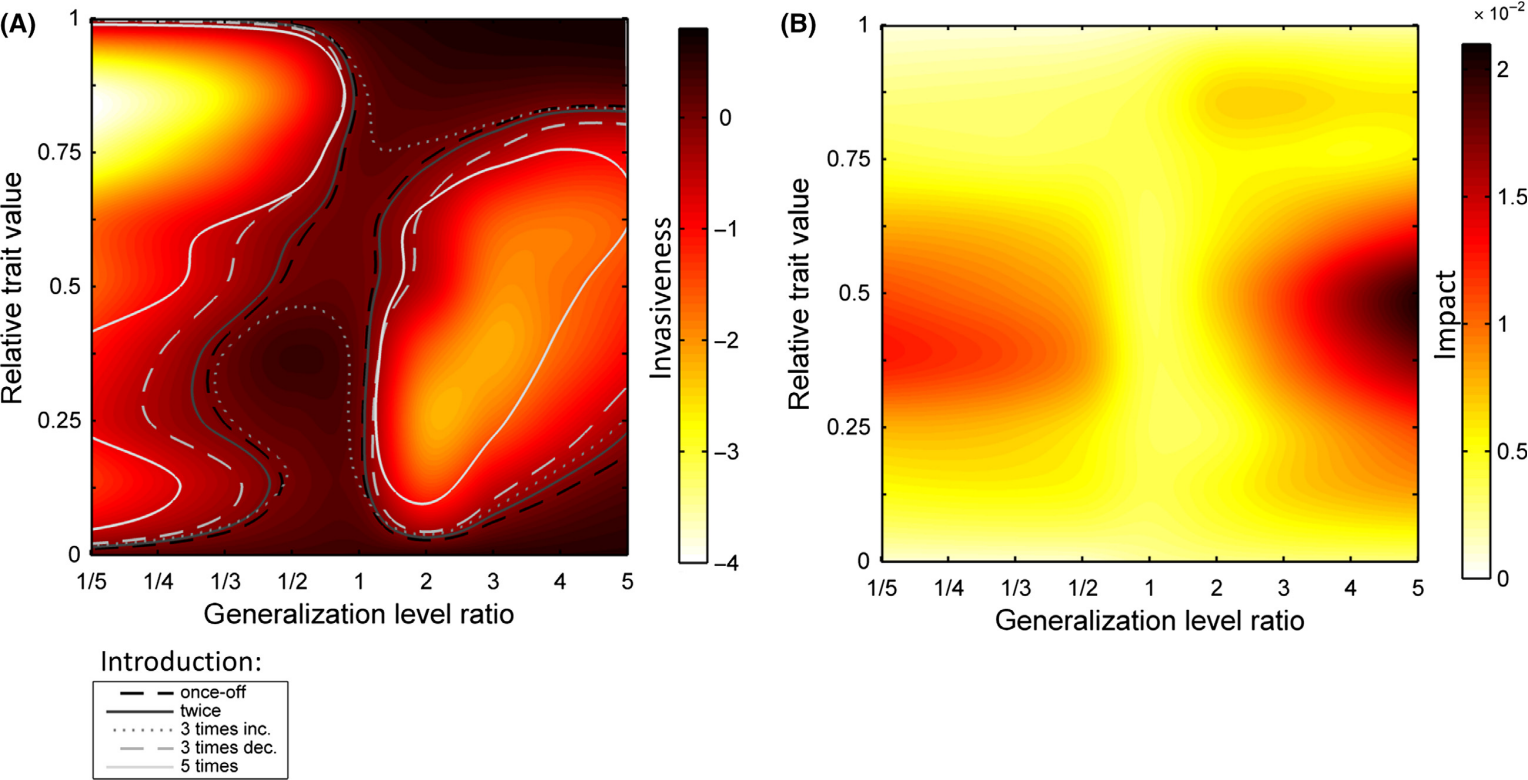
(A) Invasiveness and (B) impact of the invader as a function of its relative trait value and generalization level ratio, relative to those of the native community. Invasiveness and impact values represent the average over 100 medium‐size networks. Lines represent the zero level of invasiveness under different introduction modes.

Both the generalization level of the invader and its trait had an effect on the invasion success (Fig. 2). In general, species having the level of generalization similar to that of the natives are more likely to be invasive (vertically centered area of Fig. 2A). Species having extreme trait values but a high level of generalization also have high invasiveness (top‐right and bottom‐right corners of Fig. 2A). Species that are extreme specialist with extreme trait values also tend to be more invasive than those with trait value similar to most of the native species (extreme left area of Fig. 2A). Although the trait of the invader and its level of generalization can affect the population density of the native community, the overall impact of the invasion is small, reducing the total population size of the entire native community by about 1% (Fig. 2B). Highly generalist species having trait values similar to those of native species have the highest impact on the native community (center‐right area of Fig. 2B). The impact is also high for extreme specialist species having trait values similar to natives (center‐left area of Fig. 2B). The introduction of species having extreme trait values or having level of generalization similar to those of the natives only slightly affected native population densities (top, bottom, and vertically centered areas of Fig. 2B). Moreover, when the introduced species has a trait value that falls far outside the range of resident traits, its invasiveness and impacts become trivial because it is situated far from the resource optimum and thus suffers from the lack of resources (Fig. S1). For 89% of the studied cases, the introduction of the alien species made the total population density decline (Fig. S2).

### Role of introduction mode

**Figure 3:**
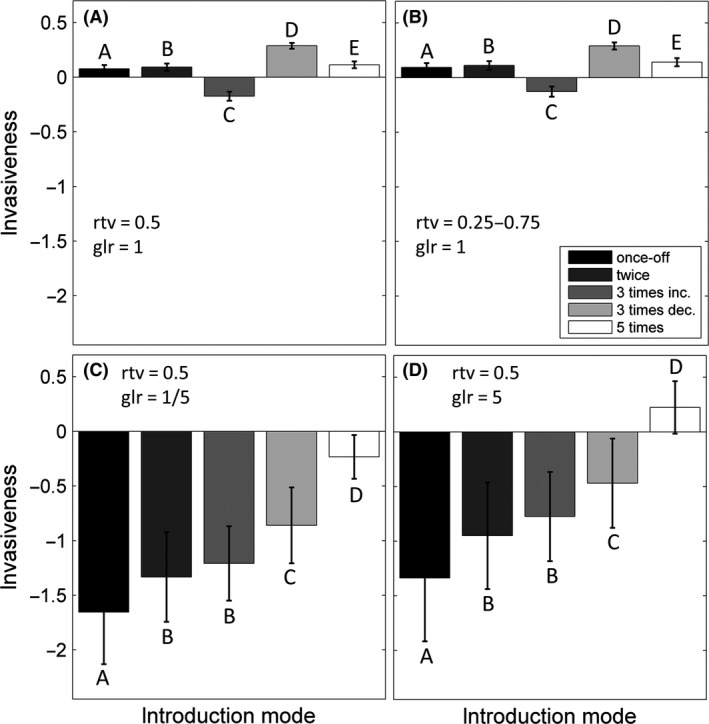
Average (over 100 medium‐size networks) of the invasiveness when the alien has the following: (A) typical trait and similar level of generalization to the native species, (B) average trait and similar level of generalization to native species, (C) typical trait and is more specialist than native species, (D) typical trait and is more generalist than native. Error bars represent tenth of the standard deviation. rtv stands for relative trait value and glr for generalization level ratio. Bars with different characters are significantly different from each other.

Invasion success also depends on the way these alien individuals are introduced (e.g., once‐off or multiple introductions), that is, the introduction mode. However, the dependence of invasiveness on introduction mode is sensitive to the level of generalization of the invader. First, when an invader has the same level of generalization as the native species, its invasiveness becomes the highest for the mode of three introductions with decreasing propagule sizes and becomes the lowest for the mode of three introductions with increasing propagule sizes (Fig. 3A and B). Second, when the invader is either more specialist or more generalist than the native species, the invasiveness of the alien becomes highly dependent on the number of introduction events, with higher numbers of introductions leading to high invasiveness (Figs. 3C and D, S3).

**Figure 4:**
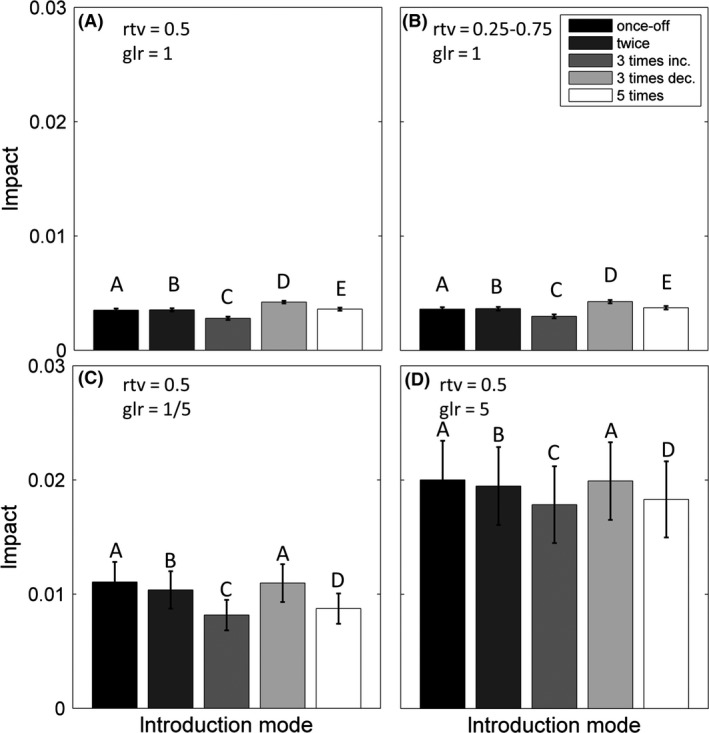
Average (over 100 medium‐size networks) of the impact when the alien has the following: (A) typical trait and similar level of generalization to the native species, (B) average trait and similar level of generalization to native species, (C) typical trait and is more specialist than native species, (D) typical trait and is more generalist than native. Error bars represent tenth of the standard deviation. rtv stands for relative trait value and glr stands for generalization level ratio. Bars with different characters are significantly different from each other.

The dependence of the invasion impact on the mode of introduction is uniform regardless of the invader trait value and its generalization level. The impact of the invasion on the population of the native community is highest when the invader is introduced three times with decreasing propagule sizes (Fig. 4). However, when the invader species is highly specialist or highly generalist, the impact of multiple introductions is not significantly different from the impacts caused by a once‐off introduction (Fig. 4C and D). Regardless of the introduction mode (Figs. S3, S4) and the initial propagule size (Fig. S5), these patterns demonstrated in the previous section regarding the dependence of invasiveness and impact on the invader trait and its level of generalization remained.

### Role of network structure and stability

**Figure 5:**
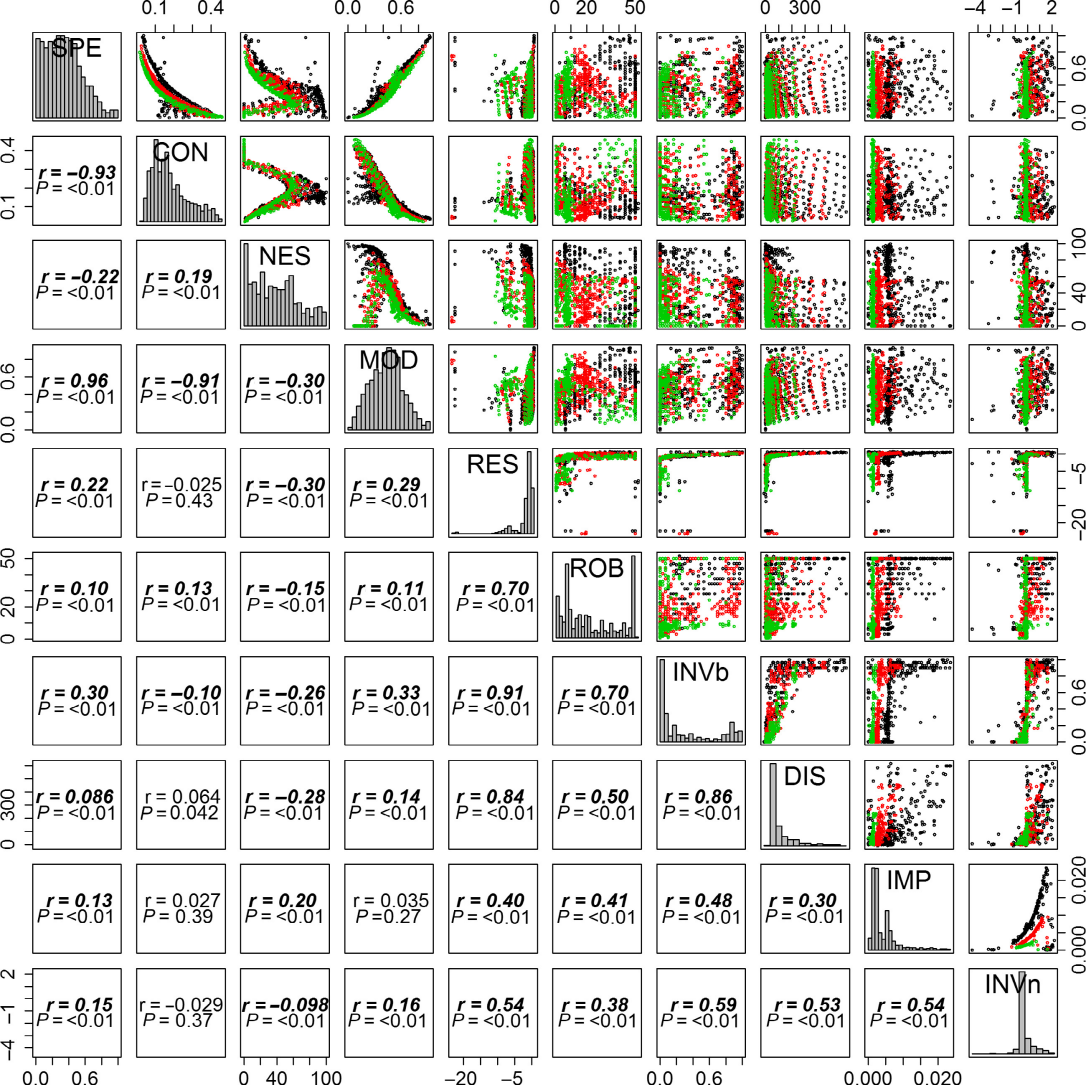
Spearman's rank correlations between network metrics. The lower triangular block gives the rank correlation coefficient (*r*) and the *P*‐values. Diagonal plots represent histograms of each network metric. Green, red, and black dots represent, respectively, small‐, medium‐, and large‐size networks.

Although most network architectural metrics had a significant relationship with network stability metrics (including invasibility), these relationships are quite weak, with the strongest being between modularity and invasibility (Spearman's rank correlation *r* = 0.33; Fig. 5). Network connectance is the weakest related to network stability yet still significant with network robustness (*r* = 0.13) and invasibility (*r* = −0.10), regardless of the initial propagule size of the invader (Figs. 5, S6). Specialization and modularity affect all network stability positively (including positively with invasibility). In contrast, nestedness is negatively correlated with most network stability metrics, except for its positive relation with invasion impact (Figs. 5, S6).

Network architectural metrics are more closely related with themselves rather than with metrics of network stability or invasibility. In particular, modularity and specialization are strongly positively correlated (*r* = 0.96), while nestedness forms a hook‐shaped relationship with other network architectural metrics. Network stability metrics are also more strongly correlated within themselves rather than with network architectural metrics. Specifically, we noticed strong positive relationships among resilience, invasibility, and disruptiveness, regardless of the initial propagule size (Figs. 5, S6). Measurement of invasion impact has the lowest correlations with metrics of network stability (Figs. 5, S6). Of particular interest, although invasibility, disruptiveness, impact, and invasiveness are conceptually measures of network instability, they are nonetheless positively correlated with network robustness and resilience. That is, the most robust and resilient community is also the one that is the most disruptive and easy to invade, suggesting the existence of two conceptually related but distinct groups in network stability metrics.

**Figure 6:**
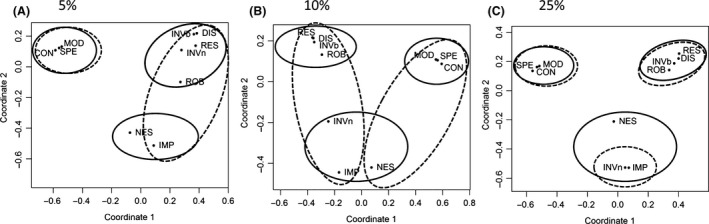
Multidimensional scaling representation of the relationship between all network metrics under different propagule sizes, in which respectively (A) 95.5%, (B) 95.2, and (C) 96.3% of the variance was explained. The number of introduced individuals is respectively (A) 5%, (B) 10%, and (C) 25% of the average native population densities. Clusters formed by the *k*‐mean clustering analysis are shown by solid circles and those from a hierarchical clustering by dashed circles.

Using multidimensional scaling analysis, we confirmed that there are two groups of metrics for network architecture and stability (Fig. 6). The *k*‐mean clustering analysis gave an optimal number of three clusters, irrespective of the propagule size, with more than 95% variance explained. There is an additional third group containing nestedness, invasion impact, and the invasiveness (Fig. 6B and C). When the initial propagule size of the invader is small (5% of the average native density), invasiveness became less related to nestedness but joined the group of network stability metrics (Fig. 6A). Results from the hierarchical clustering using a *P*‐value >0.95 confirmed once again about the two groups of network metrics, in agreement with the grouping from the *k*‐mean clustering analysis (Fig. 6). Members of the third additional group are either divided into the other two main groups or left in isolation. In particular, nestedness is generally weakly related to both main groups of network metrics (Fig. 6A and C).

## Discussion

### Trait‐mediated invasiveness and impact

Ecological network approach in which interactions are mediated by traits constitutes an interesting framework to predict the success or the failure of an invasion. It allowed us to test the invasion success for different combinations of invader characteristics (trait and level of generalization) and the characteristics of the recipient community. In contrast to previous studies (Aizen et al. 2008; Albrecht et al. 2014; Campbell et al. 2015), we found that the effect of invader characteristics on its invasion success is not unidirectional but intertwined. However, our finding that alien species with traits dissimilar to those of the natives are the most invasive ones is consistent with previous studies (Aizen et al. 2008; Campbell et al. 2015). The importance of high interaction generalization to invasiveness as observed by others (Aizen et al. 2008; Bartomeus et al. 2008; Vilà et al. 2009; Albrecht et al. 2014) was only observed in our results when the traits of the invader are dissimilar to the average resident traits. Our results, thus, encompass broader scenarios than those previously studied on mutualistic networks. The most invasive species is not always the one that has the biggest impact, highlighting the need to differentiate highly invasive species from those with big impact in management prioritization. Invasive species should only be targeted by management if their negative impacts outweigh their positive effects.

Besides trait distinctiveness, a high level of interaction generalization is also a strong predictor for big impacts (Aizen et al. 2008; Albrecht et al. 2014), often through the cascading effect of interactions that are strongly associated with generalists. Different from Campbell et al. (2015) but consistent with Morales and Traveset (2009), we found that invaders with traits atypical of the native community have the least impact to native population sizes. As the overall impact observed in our model is detrimental rather than proliferating (Fig. S2), the impact probably could have resulted from intraspecific competition in mutualistic networks, suggesting that the detrimental effect from competing with invaders has overridden the proliferation from mutualistic interactions. The impact of biological invasions on native population densities is small in mutualistic networks and thus a negligible effect on network architecture (Fig. S8). Such small impact has been previously documented (Padrón et al. 2009; Vilà et al. 2009) and can be caused by the peripheral role of the invader in the network. In particular, Albrecht et al. (2014) found that the overall number of modules in an empirical pollination network was not altered by invasion, but only that modules were more connected from the super‐generalist invaders.

The trait value and node degree (level of interaction generalization) of an invader decide its invasiveness and impact in the recipient network. Our results can be explained by the balance between two forces: the detrimental effect of competition and the beneficial effect from mutualism. While a high level of interaction generalization often means large benefits from mutualism, a trait atypical of resident species means the escape from competition. Consequently, a generalist invader also possessing traits atypical of resident species is the most invasive. By contrast, to have the highest impact on the recipient network, the invader's trait should be similar to those of an average resident species so that competition can be intensified. The invader with big impact should either be an extreme generalist so that mutualistic benefits from most resident species can be monopolized, or be an extreme specialist so that benefits from targeted mutualistic partners can be deprived.

### Propagule pressure and introduction mode

Both the number of introductions and the propagule size at each introduction matter to invasion success. Even if the dependence of invasion success on the number of introductions showed contingent patterns on the level of invader generalization, a general pattern still acknowledges the importance of multiple introductions, especially with decreasing propagule size, consistent with previous studies (Jeschke and Strayer 2006; Simberloff 2009). Indeed, a high number of introductions could help in lessening environmental stochasticity (Simberloff 2009) or rescuing the establishment of each introduction as in the phenomenon of invasion meltdown (Traveset and Richardson 2014). In our case, this is probably caused by the indirect positive effect of mutualism: once some individuals of the invader establish in the system, they proliferate the population densities of their mutualistic partners and subsequently facilitate the establishment of new arrivals from future introductions, potentially forming a positive feedback between aliens and natives in mutualistic networks (Memmott and Waser 2002; Bartomeus et al. 2008; Traveset and Richardson 2014). Moreover, the additional effect of decreasing propagule size in multiple introductions suggests that such proliferation from earlier introductions is diminishing or saturating with the number of established individuals.

### Network architecture and invasibility

Network structures, such as connectance, level of specialization, nestedness, and modularity, were shown to be not of primary correlates of network stability. Consequently, network architectures alone cannot capture the overall functioning of ecological networks. More importantly, one measure of network stability would suffice for predicting how a community responds to the perturbation of biological invasions. We are certainly not discarding the role of network architectures in stabilizing or destabilizing mutualistic networks (Bastolla et al. 2009; Thébault and Fontaine 2010; Allesina and Tang 2012; Rohr et al. 2014; Vieira and Almeida‐Neto 2015), but simply state that inferring network function from structure could have been overemphasized. In particular, nestedness was negatively correlated with resilience and robustness, consistent with previous studies (Allesina and Tang 2012; Campbell et al. 2012), even though it has been observed as one of the most prominent characteristics of mutualistic networks. This counter‐intuitive observation is reconciled by our results that highly nested networks have a low invasibility, thus less likely to be invaded.

The more robust and resilient a network is, the more susceptible it is to invasion. Mutualistic networks which are well posed (high robustness) can return quickly to a steady state after perturbations (high resilience); such network features also make it susceptible to invasion (high invasibility; i.e., a high chance of invasion success). Intuitively, this is because the features of a network being well posed also allow it to easily absorb newly introduced species. That is, networks that are insensitive to perturbations, especially to species removal (i.e., being robust) will have a high chance to be invaded. The positive relationships between network stability metrics (resilience and robustness) and network instability metrics (invasibility, invasiveness, disruptiveness, and impact) heighten the necessity to use appropriate measures in network studies. Stability metrics should therefore not be interpreted outside the context defining environmental drivers of change (Ives and Carpenter 2007). Moreover, network resilience and disruptiveness are strongly related to each other (Fig. 6). As the former is widely used as a proxy of ecological stability and the latter evolutionary instability, resilient networks are disruptive. Ecological stability and evolutionary stability could be two complementary states for systems to handle perturbations.

Future works can expand the scope of our model in two aspects. First, although we were able to vary the interaction generalization level of the invader, the levels of interaction generalization of all native species were assumed to be the same (i.e., the tolerance to trait difference *σ*_*m*_). This assumption could have oversimplified the reality that species in real networks often have different diet breadths. Second, we assumed a symmetric model regarding the animal–plant interaction. Empirical studies have often unveiled imbalanced roles of animal pollinators and flowering plants in mutualistic networks, resulting in asymmetric interaction with plants strongly dependent on the pollinators (Bascompte et al. 2006; Aizen et al. 2008). Extension of our trait‐based model to encompass interaction asymmetry would certainly be worth of further investigation.

## Conflict of Interest

None declared.

## Supporting information


**Figure S1.** (a) Invasiveness and (b) impact (averaged over 100 medium‐size networks) of the invader as a function of invader characteristics when the invader is introduced once. Traits of the alien species can be outside the range of resident traits. White lines represent the zero level of invasiveness.
**Figure S2.** Relative growth rate of the native species (average over 100 medium‐size networks) as a function of the invader characteristics for once‐off introduction. The white line near the bottom right represents the zero growth line.
**Figure S3**. Invasiveness (average over 100 medium‐size networks) as a function of invader characteristics when introduced (a) twice with equal propagule sizes, (b) three times with increasing propagule sizes, (c) three times with decreasing propagule sizes and (d) five times with equal propagule sizes. White lines represent the zero invasiveness.
**Figure S4**. Impact (average over 100 medium‐size networks) as a function of invader characteristics when introduced (a) twice with equal propagule sizes, (b) three times with increasing propagule sizes, (c) three times with decreasing propagule sizes and (d) five times with equal propagule sizes.
**Figure S5**. Invasiveness (a, b) and impact (c, d), average over 100 medium‐size networks, as a function of invader characteristics when introduced once‐off, under different initial propagule sizes. (a) and (c): 5% of the average native density; (b) and (d): 25% of the average native density. White line represents the zero invasiveness.
**Figure S6.** Relationships between all network metrics for different initial propagule sizes. The lower triangular block contains the Spearman's rank correlation coefficient (*r*) and the *P*‐values. Diagonal plots represent histograms of each network metrics. Green, red and black dots represent, respectively, small‐, medium‐, and large‐size networks.
**Figure S7.** Relationships between all network metrics for different network sizes. The lower triangular block contains the Spearman's rank correlation coefficient (*r*) and the *P*‐values. Diagonal plots represent histograms of each of the network metrics. Green, red and black dots represent, respectively, small‐, medium‐, and large‐size networks.
**Figure S8.** Comparison of network architectures between pre‐ and postinvasion networks. Points represent the average values over all networks. Error bars are standard deviations. Green, red and black colors represent, respectively, small‐, medium‐, and large‐size networks.
