# Supplementary material for: Invading a mutualistic network: to be or not to be similar
Source: Ecol Evol. 2016 Jun 23;6(14):4981–96. doi: 10.1002/ece3.2263 (PMC4979722; doi:10.1002/ece3.2263)
Supplement: Supplementary file 1 — Figure S1. (a) Invasiveness and (b) impact (averaged over 100 medium‐size networks) of the invader as a function of invader characteristics when the invader is introduced once. Traits of the alien species can be outside the range of resident traits. White lines represent the zero level of invasiveness. Figure S2. Relative growth rate of the native species (average over 100 medium‐size networks) as a function of the invader characteristics for once‐off introduction. The white line near the bottom right represents the zero growth line. Figure S3. Invasiveness (average over 100 medium‐size networks) as a function of invader characteristics when introduced (a) twice with equal propagule sizes, (b) three times with increasing propagule sizes, (c) three times with decreasing propagule sizes and (d) five times with equal propagule sizes. White lines represent the zero invasiveness. Figure S4. Impact (average over 100 medium‐size networks) as a function of invader characteristics when introduced (a) twice with equal propagule sizes, (b) three times with increasing propagule sizes, (c) three times with decreasing propagule sizes and (d) five times with equal propagule sizes. Figure S5. Invasiveness (a, b) and impact (c, d), average over 100 medium‐size networks, as a function of invader characteristics when introduced once‐off, under different initial propagule sizes. (a) and (c): 5% of the average native density; (b) and (d): 25% of the average native density. White line represents the zero invasiveness. Figure S6. Relationships between all network metrics for different initial propagule sizes. The lower triangular block contains the Spearman's rank correlation coefficient (r) and the P‐values. Diagonal plots represent histograms of each network metrics. Green, red and black dots represent, respectively, small‐, medium‐, and large‐size networks. Figure S7. Relationships between all network metrics for different network sizes. The lower triangular block contains the Spearman [file ECE3-6-4981-s001.docx]

**Supplementary materials**

H. Onivola Minoarivelo & Cang Hui. Invading a mutualistic network: to be or not to be similar.

| ****  **Figure S1**: (a) Invasiveness and (b) impact (averaged over 100 medium size networks) of the invader as a function of invader characteristics when the invader is introduced once. Traits of the alien species can be outside the range of resident traits. White lines represent the zero level of invasiveness. |
| --- |

| ****  **Figure S2:** Relative growth rate of the native species (average over 100 medium size networks) as a function of the invader characteristics for once-off introduction. The white line near the bottom right represents the zero growth line. |
| --- |

| ****  **Figure S3**: Invasiveness (average over 100 medium size networks) as a function of invader characteristics when introduced (a) twice with equal propagule sizes, (b) three times with increasing propagule sizes, (c) three times with decreasing propagule sizes and (d) five times with equal propagule sizes. White lines represent the zero invasiveness. |  |
| --- | --- |
| ****  **Figure S4**: Impact (average over 100 medium size networks) as a function of invader characteristics when introduced (a) twice with equal propagule sizes, (b) three times with increasing propagule sizes, (c) three times with decreasing propagule sizes and (d) five times with equal propagule sizes. | |

| ****  **Figure S5**: Invasiveness (a, b) and impact (c, d), average over 100 medium size networks, as a function of invader characteristics when introduced once-off, under different initial propagule sizes. (a) and (c): 5% of the average native density; (b) and (d): 25% of the average native density. White line represents the zero invasiveness. |
| --- |

**Figure S6:** Relationships between all network metrics for different initial propagule sizes. The lower triangular block contains the Spearman’s rank correlation coefficient (r) and the p-values. Diagonal plots represent histograms of each network metrics. Green, red and black dots represent respectively small, medium and large size networks.

**initial propagule size = 5%**

**initial propagule size = 25%**

**Figure S7:** Relationships between all network metrics for different network sizes. The lower triangular block contains the Spearman’s rank correlation coefficient (r) and the p-values. Diagonal plots represent histograms of each of the network metrics. Green, red and black dots represent respectively small, medium and large size networks.

**small networks**

**medium-size networks**

**large networks**

**Figure S8**: Comparison of network architectures between pre- and post-invasion networks. Points represent the average values over all networks. Error bars are standard deviations. Green, red and black colours represent respectively small, medium and large size networks.
